# The Efficacy of Plant-Based Ionizers in Removing Aerosol for COVID-19 Mitigation

**DOI:** 10.34133/2021/2173642

**Published:** 2021-02-11

**Authors:** Ady Suwardi, Chin Chun Ooi, Dan Daniel, Chee Kiang Ivan Tan, Hongying Li, Ou Yang Zhong Liang, Yuanting Karen Tang, Jing Yee Chee, Anton Sadovoy, Shu-Ye Jiang, Srinivasan Ramachandran, Enyi Ye, Chang Wei Kang, Wun Chet Davy Cheong, Keng Hui Lim, Xian Jun Loh

**Affiliations:** ^1^Institute of Materials Research and Engineering, 2 Fusionopolis Way, Agency for Science, Technology and Research, Singapore 138634; ^2^Institute of High Performance Computing, 1 Fusionopolis Way, Agency for Science, Technology and Research, Singapore 138632; ^3^Temasek Life Sciences Laboratory, 1 Research Link, National University of Singapore, Singapore 117604

## Abstract

Small-sized droplets/aerosol transmission is one of the factors responsible for the spread of COVID-19, in addition to large droplets and surface contamination (fomites). While large droplets and surface contamination can be relatively easier to deal with (i.e., using mask and proper hygiene measures), aerosol presents a different challenge due to their ability to remain airborne for a long time. This calls for mitigation solutions that can rapidly eliminate the airborne aerosol. Pre-COVID-19, air ionizers have been touted as effective tools to eliminate small particulates. In this work, we sought to evaluate the efficacy of a novel plant-based ionizer in eliminating aerosol. It was found that factors such as the ion concentration, humidity, and ventilation can drastically affect the efficacy of aerosol removal. The aerosol removal rate was quantified in terms of ACH (air changes per hour) and CADR- (clean air delivery rate-) equivalent unit, with ACH as high as 12 and CADR as high as 141 ft^3^/minute being achieved by a plant-based ionizer in a small isolated room. This work provides an important and timely guidance on the effective deployment of ionizers in minimizing the risk of COVID-19 spread via airborne aerosol, especially in a poorly-ventilated environment.

## 1. Introduction

SARS-COV-2/COVID-19 has resulted in tremendous loss of lives and adversely impacted economies globally. As of December 2020, barely a year after the first cases were reported, more than 75 million people across the world have been infected, with nearly 1.7 million fatalities [1]. Recently, North America and Europe have seen a drastic increase in both the number of new cases and fatalities, adding more than 2 million new cases and 50 thousand new fatalities in a week. This deteriorating situation can be attributed to a multitude of factors such as pandemic fatigue, lack of social distancing, and a poor adherence to mask wearing [2–6]. This worrying development is exacerbated by the winter season in the northern hemisphere where most of the world population resides (i.e., increasing the tendency for people to stay indoor during winter) [7, 8]. Therefore, it is important to identify effective mitigation measures to minimize the virus spread in poorly ventilated indoor environments.

Based on the latest findings, The Centers for Disease Control and Prevention (CDC) has categorized the COVID-19 transmission pathways into three modes, namely, contact transmission (fomites), droplets transmission, and airborne (aerosol) transmission [9]. To mitigate the effects of COVID-19, countries have implemented different measures such as various degrees of lockdown, contact tracing, and social distancing measures [10–12]. When strict enforcements are in place, these measures are effective in minimizing contact and droplets transmission. This was evidenced by the rapidly declining number of cases during the early days of the pandemic in countries with relatively stringent measures [10, 13]. However, unlike contact and large droplets transmission, aerosol can stay suspended in the air for hours and travel over long distances, which present great challenges in mitigation and contact tracing [9, 14]. To date, the understanding of airborne (aerosol) transmission and the effectiveness of appropriate mitigation measures remain unclear [15]. This is not to mention that there is a lack of standardization in particulate matters and bioaerosol sampling techniques [16].

In order to minimize virus spread through aerosol transmission, engineering controls such as ventilation, particle filtration, air disinfection, masks, and engineering air circulation can be used as a low-cost yet effective approach [17–23]. However, some of these measures can be challenging to be implemented across widely varying social settings. While ventilation has been proven to be effective in indoor settings, it cannot be implemented in all places and environmental conditions. These include indoor venues without air-exchangers or where the opening of windows is impractical. In addition, air-disinfection by ultraviolet light has been proven to be effective, but can present negative effects to human health [18]. Finding a readily deployable solution to effectively control and reduce aerosol concentration in a poorly ventilated indoor setting is therefore highly desirable.

Previously, ionizers have been shown to reduce small particulate concentration, airborne bacterial levels, and common influenza virus infectivity [24–26]. Ionizers typically produce negative air ions (NAIs), which are unipolar ions that can electrically charge particulate matter (PM). PM can be efficiently removed in the environment with a high concentration of NAIs, as the charged PM is attracted to nearby surfaces and can settle down more quickly [27–31]. However, a major disadvantage of many ionizers is the emission of ozone, which is harmful to human health [32–34].

Plant-based ionizer has been shown to generate a high concentration of NAIs (up to 82 million ions/cm^3^) under pulsed electrical field (PEF) conditions while producing no ozone [35]. Plant-based ionizers have been shown to effectively remove PM_2.5_ level in an enclosed chamber (from around 500 to near 0 *μ*g/m^3^ within 5 minutes) [35].

In this study, we sought to evaluate the efficacy of plant-based ionizers in reducing the aerosol concentration in a poorly ventilated indoor setting. Different parameters such as the number and size of plant-based ionizers, humidity, and ventilation were varied to evaluate their effects on aerosol removal. To enable intuitive comparison, we quantified the efficacy of the plant-based ionizers in terms of ACH- (air changes per hour-) equivalent. This is useful as ACH is a simple and intuitive metric for evaluating the effectiveness of ventilation in the design of indoor spaces. It is worth noting that the ACH-equivalent value here does not refer to the real air-exchange in the room. Instead, it represents the equivalent aerosol removal rate as a room with the same air exchange rate.

This ACH-equivalent value can also be expressed in terms of CADR- (clean air delivery rate-) equivalent, which is traditionally used to quantify the contaminant removal capacity for air purifiers. Remarkably, in a poorly ventilated 20 m^3^ room with 1 large plant-based ionizer (SP4000), 95% aerosol reduction was achieved within 7 minutes, which is equivalent to the effect of having ACH of 12 and CADR of 141 ft^3^/minute. In contrast, the recommended minimum ventilation according to CDC (centers for disease control and prevention) guidelines is 6 ACH. Lastly, it was found that humidity plays an important role in the rate of aerosol removal with or without plant-based ionizers. The findings from this work can be used to guide the effective deployment of plant-based ionizers in poorly ventilated indoor environment.

## 2. Methodology

### 2.1. Experimental

**Figure 1:**
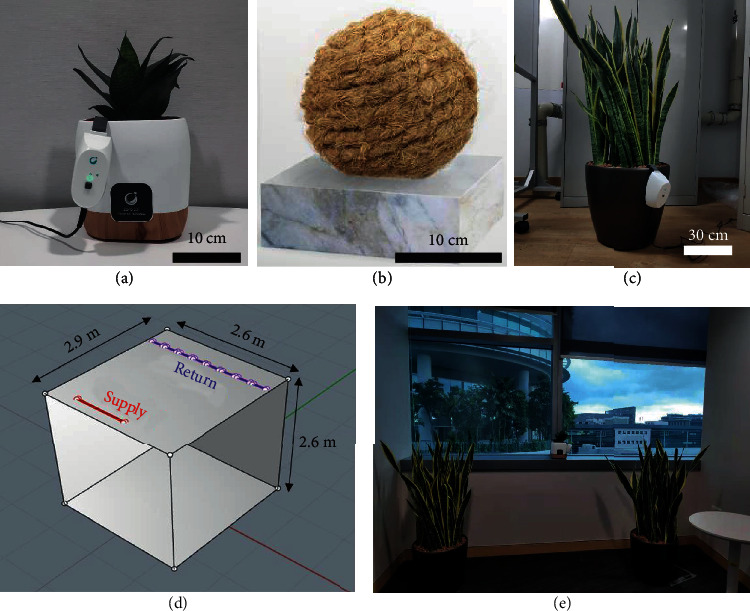
Experimental setup showing (a) SP2800-small plant-based ionizer. (b) Coco coir ball shape ionizer. (c) SP4000-large plant-based ionizer. (d) Layout of the small office showing the dimension, inlet, and outlet for air exchange. (e) Actual room used for experiments.

Commercially-available plant-based ionizers from Zero2.5 Biotech Pte. Ltd. (model SP2800-denoted as small plant-based ionizers; model CF4000-denoted as coco coir; and model SP4000-denoted as large plant-based ionizer hereon) were used in this study (Figure 1(a)–(c)). The estimated NAIs at 1 m from SP2800 and SP4000 are 200000-400000 and 1 million ions/cm^3^, respectively (https://negativeairion.com/). As a control, we also compare the efficacy of plant-based ionizers with commercial ionizers. In order to simulate aerosol generated by a human subject, LED-500 fogging machine was used to generate aerosol with a mean particle size of approximately 1 *μ*m in diameter. A mixture of 50% water and 50% glycerol was used as the fogging liquid. Particulate sensors (Sensirion SPS30) were used to detect and quantify the aerosol concentration. In all experiments, the fog machine was used to saturate the room/enclosure with aerosol (Figure 1(e)), aided by a small fan, until the reading in multiple particle sensors across the room shows similar and consistent values. The dimensions of the poorly ventilated room are shown in Figure 1(d). The aerosol concentration was continuously measured with each of the sensors making a measurement every second, with real-time data sent wirelessly to a computer that logs the data. The sensors were calibrated using TSI DustTrak™ DRX Aerosol Monitor 8533 as a reference. To ensure data accuracy, the linearity of the particle sensor was tested and calibrated for 1 *μ*m size aerosol.

### 2.2. Theoretical Modeling

In order to have an intuitive comparison between the efficiency of plant-based ionizers and the air exchange in a space, we have adopted computational fluid dynamics (CFD) to simulate fluid dynamics as well as the particle movement under different ACH. A generic indoor space of dimensions 2.6 m (length) × 2.8 m (width) × 2.8 m (height) is modelled, with inlet and outlet slots at the two ends of the ceiling, similar to that of the experimental venue (as in Figure 1(e)). The computer model is illustrated in Figure 1(d). Meshing is conducted using ANSYS FLUENT 2019 via the mosaic meshing scheme. The minimum cell size used is 0.1 mm while the maximum cell size is of length 0.1 m, resulting in a total mesh size of 2.04 million cells. The room is assumed to be empty with no furniture or objects in the room. An operating temperature of 26°C is specified for consistency with the experiments. Inlet and outlet flow velocities are adjusted to provide air changes per hour (ACH) within the room ranging from ACH = 1 to ACH = 15 for this study. This corresponds to inlet velocities between 0.14 m/s and 2.1 m/s, respectively.

The particles are assumed to be perfectly spherical particles of 1 *μ*m diameter, in accordance with the experiments. The particles are specified with a density of 1130 kg/m^3^, which corresponds to 50% glycerol and water by mass. This value is close to the density of human saliva (1012 kg/m^3^) [36]. The inclusion of glycerol in water generally reduces water activity which in turn hinders droplet evaporation. With 50% glycerol in water, it was previously reported that the glycerol-water droplet will have minimal evaporation in an environment with RH = 0.66; hence, evaporation is neglected in the numerical simulations [37]. The simulated particles are also assumed to be noninteracting in this set of simulations due to the low particle concentration (<1 ppm).

For all particle simulations, a steady-state simulation is first performed to obtain a converged solution for fluid flow within the domain. The steady-state solution is then used as input for the simulation of particle trajectories based on the discrete phase model. 10^4^ particles are randomly dispersed in the room at the start of each particle simulation, and individual particle trajectories are tracked until the particles either exit the room through the exhaust vents or are trapped on the room's surfaces. In these simulations, all surfaces in the domain are assumed to be perfect traps for the particles, i.e., particles are assumed to be trapped upon contact with the walls, floor, or ceiling. Similarly to the experiments, the fraction of particles left afloat in the room is tracked with time, and a corresponding decay time constant is calculated.

### 2.3. Fluid Dynamics and Heat Transfer

The governing equations for fluid mass and momentum with turbulence are

(1) $$\begin{array}{c} \frac{\partial \rho }{\partial t}+\nabla \cdot \left(\rho \vec{u}\right)=\dot{m,} \end{array}$$

(2) $$\begin{array}{c} \frac{\partial \left(\rho \vec{u}\right)}{\partial t}+\nabla \cdot \left(\rho \vec{u}\vec{u}\right)=-\nabla P+\nabla \cdot \left[\left(\mu +\mu_{t}\right)\left(\nabla \vec{u}+\nabla {\vec{u}}^{T}\right)\right]-\nabla \cdot \left(\frac{2}{3}\rho \kappa \vec{I}\right)+F_{m}, \end{array}$$

where *κ* is the turbulent kinetic energy and *ε* is the dissipation of turbulent energy, expressed as

(3) $$\begin{array}{c} \frac{\partial \left(\rho \kappa \right)}{\partial t}+\nabla \cdot \left(\rho \vec{u}\kappa \right)=\nabla \cdot \left(\frac{\mu_{t}}{\sigma_{k}}\nabla \kappa \right)+G_{k}-\rho \varepsilon , \end{array}$$

(4) $$\begin{array}{c} \frac{\partial \left(\rho \varepsilon \right)}{\partial t}+\nabla \cdot \left(\rho \vec{u}\varepsilon \right)=\nabla \cdot \left(\frac{\mu_{t}}{\sigma_{\varepsilon }}\nabla \varepsilon \right)+\frac{\varepsilon }{\kappa }\left(C_{1\varepsilon }G_{k}-C_{2\varepsilon }\rho \varepsilon \right), \end{array}$$

where *C*_1*ε*_ and *C*_2*ε*_ are constants 1.44 and 1.92, respectively, *σ*_*κ*_ and *σ*_*ε*_ are 1.00 and 1.3, respectively. *G*_*k*_ is the production of turbulence kinetic energy.

Eddy viscosity *μ*_*τ*_ is expressed as

(5) $$\begin{array}{c} \mu_{t}=\rho C_{\mu }\frac{\kappa^{2}}{\varepsilon }, \end{array}$$

where *C*_*μ*_ is equal to 0.09.

In this work, the realizable *k* − *ε* model is used for the modeling of turbulence.

### 2.4. Droplet Tracking Model

The equation of motion of a droplet (subscript *d*) is

(6) $$\begin{array}{c} \frac{d{\vec{u}}_{d}}{dt}=-F_{D}\left({\vec{u}}_{d}-\vec{u}\right)+\frac{\vec{g}\left(\rho_{d}-\rho \right)}{\rho_{d}}. \end{array}$$

${\vec{u}}_{d}$ and $\vec{u}$ are the droplet and air velocities, respectively. *F*_*D*_ is the drag force,

(7) FD=18μρdDd2CDRe24,

where *D*_*d*_ is the droplet diameter and *C*_*D*_ is the drag coefficient as a function of the droplet Reynolds number,

(8) CD=c1+c2Re+c3Re2,

(9) Re=ρDdu→d−u→μ,

where *c*_1_, *c*_2_, and *c*_3_ are empirical constants for spherical droplets which vary with Reynolds number as follows:

(10) c1,c2,c3=0,24,0  0<Re<0.1,3.69,22.73,0.0903  0.1<Re<1,1.222,1.667,−3.889  1<Re<10,0.6167,46.5,−116.67  10<Re<100,0.3644,98.33,−2778  100<Re<1000,0.357,148.62,−47500  1000<Re<5000,0.46,−490.546,578700  5000<Re<10000,0.5191,−1662.55416700  Re>10000.

### 2.5. Particle Decay Model

The decay of particles within the room is approximated to first order by the following equation:

(11) $$\begin{array}{c} C\left(t\right)=C_{o}e^{-t\tau }, \end{array}$$

where *C* is the particle concentration, *C*_*o*_ is the particle concentration when *t* = 0, and *τ* is a characteristic decay constant. *τ* is a variable, which will vary with different ventilation rates within the indoor space, presence of different plant-based ionizers, and likelihood of the particle to stick to various indoor surfaces upon contact. This is approximated from the rate of decay of particle concentration as measured in the experiments and from the rate of decay of a randomly distributed set of 10^4^ particles in the room in the numerical simulations across different ventilation settings.

### 2.6. ACH and CADR

To obtain the CADR-equivalent value from an ACH-equivalent in a room with known dimension, the following equation can be applied:

(12) $$\begin{array}{c} \text{CADR}=\text{ACHx}V*\frac{35.315}{60}, \end{array}$$

where CADR is the clean air delivery rate (ft^3^/minute), *V* is the room volume (m^3^), and ACH is air change per hour.

## 3. Results and Discussion

**Figure 2:**
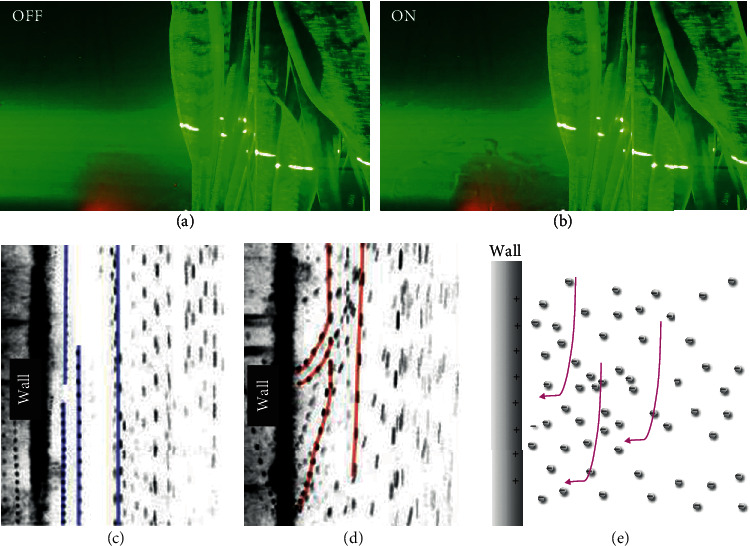
Laser sheet visualization images of the plant-based ionizers showing contrasting behaviour of aerosol around the plant-based ionizer when it is turned (a) off and (b) on, which shows the swirling effect generated by the charged particles around the large plant-based ionizers. High-speed camera image showing the aerosol movement around the wall of an enclosure when the plant-based ionizer was turned (c) off and (d) on. (e) Graphical illustration of charged aerosol settling inducing opposite charges and being deposited on the wall.

Figure 2 shows the laser sheet visualization images which show the behaviour of aerosol around the plant-based ionizer when it is turned off (Figure 2(a)) and on (Figure 2(b)). When the plant-based ionizer is turned on, swirl eddies can be observed around the plant-based ionizer (supplementary movie 1). This points to the air movement due to electrostatic interaction between electrically-charged particles/aerosol around the plant, which helps to accelerate the rate of airborne aerosol settling into fomites on the nearby surfaces. The particles/aerosol is charged due to the negative air ions (NAIs) originated from the plant-based ionizer. Figure 2(c) and (d) shows the direction of aerosol movement near to the wall when the plant-based ionizer is turned off and on, respectively. In the absence of a plant-based ionizer, aerosol travels downward due to gravitational settling. When the plant-based ionizer was turned on, the NAIs charged the aerosol, which induce opposite charges on the nearby surface/wall and consequently being deposited onto the wall as fomites (supplementary video 2). This is graphically illustrated in Figure 2(e).

In order to quantify the efficacy of the NAIs in removing the aerosol, the enclosure was saturated to a target aerosol concentration. In this study, we measured the concentration of the 1 *μ*m sized particles in the aerosol. This size is within the range of the average droplet nuclei size ranging from 0.74–2.12 *μ*m generated during a cough [38]. Our sensors were calibrated to have linear and consistent response up to 5000 particles/cm^3^. Therefore, for all experiments, only data below 4000 particles/cm^3^ was used for determining the aerosol decay rate. In addition, various experimental parameters such as the number and size of plants, temperature, humidity, and ventilation were carefully controlled and varied to isolate and reveal the intrinsic efficacy of the plant-based ionizers.

### 3.1. Number of Plant-Based Ionizers

**Figure 3:**
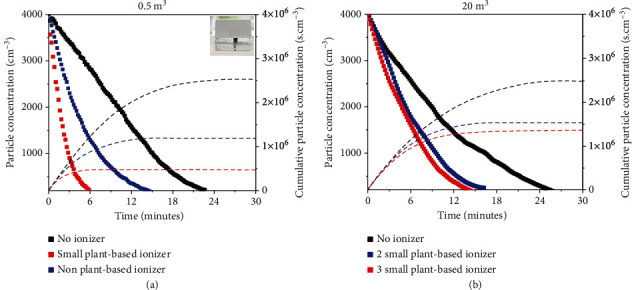
Aerosol concentration vs. time profile as a function of the number of small plant-based ionizers in (a) 0.5 m^3^ volume enclosure. (b) 20 m^3^ volume room.

In all experiments, the temperature and humidity was set at 26.0 ± 0.5°C and 66%, respectively, to ensure consistency. Data were collected over a 30-minute period when the aerosol concentration reached 4000 particles/cm^3^.  Figure 3(a) shows the aerosol concentration vs. time profile as a function of different types of ionizers in a room with a volume of 0.5 m^3^. Evidently, without any ionizer, it takes around 24 minutes for the aerosol concentration to reduce by 95%. In the absence of ionizer and ventilation, the removal of human-generated aerosol can be attributed to gravitational settling, which depends on aerosol composition and size [39–41]. Turning on a commercial ionizer (inset in Figure 3(a)) reduces the aerosol removal time to 15 minutes. When a small plant-based ionizer was turned on, it shows high efficacy in a 0.5 m^3^ enclosure in reducing the time taken to clear the aerosol from 24 minutes to 6 minutes. This is significant considering the almost five-fold difference in cumulative particle concentration over the same time period, as shown in the dashed line in Figure 3(a).

In contrast, for 20 m^3^ enclosure, no substantial effect was observed when 1 small plant-based ionizer was turned on (not shown). This may be due to the limited coverage volume for each plant. When 2 small plant-based ionizers were turned on (Figure 3(b)), the 95% aerosol removal was achieved in slightly over 15 minutes, which is almost 10 minutes faster compared to the removal time without the plant-based ionizer. Lastly, while adding a third plant does decrease the aerosol removal time to slightly below 15 minutes, it is of no substantial improvements as compared to 2 small plant-based ionizers. This suggests that the aerosol removal mechanism by the NAIs is nonlinear, as illustrated by the dashed line which shows the cumulative particle concentration. Furthermore, since the onset for effective aerosol removal in a 20 m^3^ volume room was observed when 2 small plant-based ionizers were turned on, it can be simplistically estimated that each small plant-based ionizer has a coverage volume of approximately 10 m^3^.

### 3.2. Size of Plant-Based Ionizers

**Figure 4:**
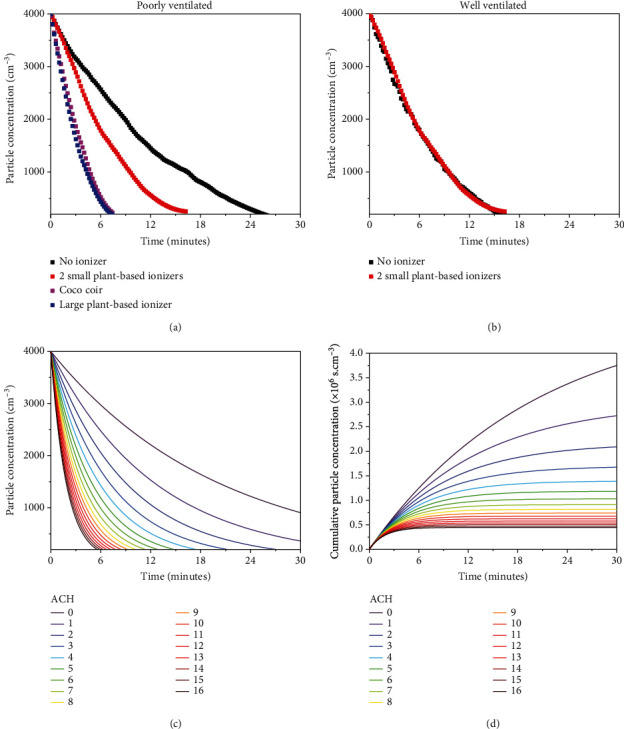
Aerosol concentration vs. time profile for small and large plant-based ionizers in (a) poorly ventilated room, (b) well-ventilated room, (c) particle concentration time decay profile for different ACH (air change per hour), and (d) cumulative particle concentration corresponding to different ACH values.

In order to examine the efficacy of large size plant-based ionizers compared to small ones, the same set of experiments was conducted in the same temperature and humidity. As shown in Figure 4(a), for a poorly ventilated room, a large plant-based ionizer drastically reduces the aerosol removal time from 25 minutes to just above 6 minutes, which is more effective compared to 2 small plants which takes 14 minutes to achieve the same removal. This observation shows that the size of plant-based ionizer arguably plays an important role in addition to the number of plants. In contrast to the coverage volume of 10 m^3^ for small plants, this observation suggests that a large plant has a coverage volume of at least 20 m^3^. The difference in efficacy between large and small plant-based ionizers can be associated to the difference in total surface area of the leaves and the voltage. Large plants have ~10 times larger leaves area and higher voltage (up to 20 kV) compared to small plants (up to 7 kV). Thus, the amount of NAIs produced in large plants is far more than those of the smaller plants [35].

In addition to the size and number of plants, ventilation plays a vital role in aerosol removal. Figure 4(b) shows the negligible effect of plant-based ionizers in a well-ventilated room (ACH > 14). In other words, plant-based ionizers can be used to create an ACH-equivalent environment when there is no possible means of natural ventilation. These are useful in situations where there is a split-type air conditioning system or the opening of windows is not possible [42]. It is worth noting that while adding extra plant-based ionizer results in diminishing additional benefit (Figure 3(b)), the decay profile from numerical simulations corresponding to different ACH numbers has a similar nonlinear nature, which is shown in Figure 4(c) and (d). Remarkably, using 3 small plant-based ionizers or a large plant-based ionizer show equivalent effects of adding up to 4 and 12 ACH to the poorly ventilated room, respectively, as summarized in Table 1.

### 3.3. Coco Coir

Besides using living plants as the plant-based ionizers, plant/fiber-based coco coir shows a similar level of efficacy as a large plant-based ionizer (Figure 4(a)). This is despite the much smaller size of the coco coir (15 cm) compared to large plant (1-meter height). The high efficacy of can be attributed to the high level of NAIs produced by the coco coir (Table 2). Structurally, the origin of high NAIs concentration is associated with the high number of sharp fibers in the coco coir, thus, concentrating higher electric field and generate more ions.

**Figure 5:**
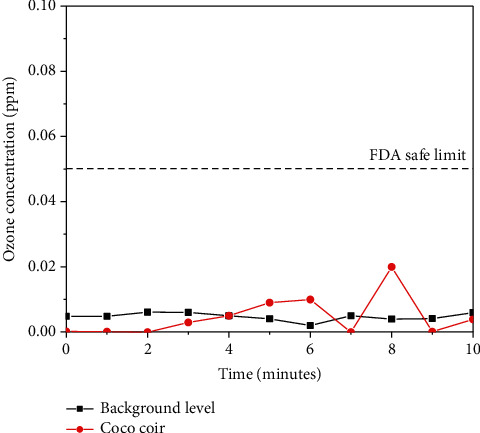
Ozone concentration measurement showing no substantial difference between the background level and coco coir.

In addition, despite the high level of NAIs, ozone concentration measurement of the coco coir shows no substantial difference to the background ozone level. Both the background and coco coir ozone levels are well within the safe limit by FDA (food and drug administration), as shown in Figure 5.

### 3.4. Humidity Level and Ventilation

**Figure 6:**
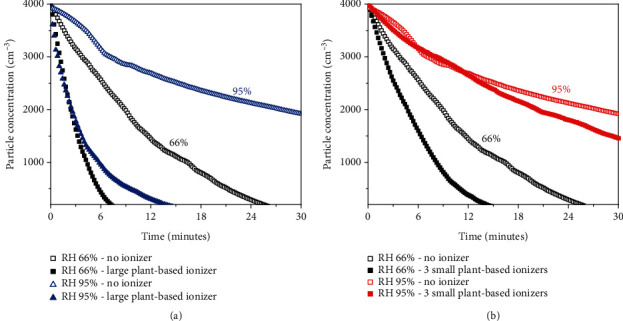
Aerosol concentration vs. time profile as a function of different relative humidity level (RH) for (a) large plant-based ionizer and (b) small plant-based ionizers.

One critical factor that is often neglected in evaluating aerosol and air quality is humidity. As a matter of fact, lower humidity has been reported to lower indoor VOCs (volatile organic compounds) concentration [43]. In this work, the effect of humidity was studied and analyzed by setting and maintaining two different relative humidity (RH) levels: 66% and 95%, respectively. As shown in Figure 6(a), in a poorly ventilated indoor setting with high humidity (RH 95%), the aerosol tends to stay afloat in the air for a very long time. In addition, even with the aid of a large plant-based ionizer, it still takes up to 14 minutes for the aerosol level to be reduced by 95%. This is twice the time taken for the same plant-based ionizer to remove 95% aerosol (7 minutes). More interestingly, in a very humid environment, small plant-based ionizers are not effective in accelerating the aerosol removal, as shown in Figure 6(b).

These observations can be associated with the competing effect brought about by floating water molecules in aerosol removal by NAIs. Therefore, for plant-based ionizers to work effectively, the humidity level has to be closely monitored. In addition, it is important to caution that while low humidity favors more effective aerosol/contaminants removal, it has also been reported that low humidity results in more aerosol generated by human subjects [44]. Thus, in the context of mitigating airborne COVID-19 transmission, further careful study has to be carried out to determine the overarching effect of humidity. It is worth noting that air circulation may help to homogenize the NAI concentration within the room, and therefore in places with air-circulation, the location/placement of the ionizer does not affect its efficacy.

### 3.5. Experimental Decay Constants

The decay of particles for the various experiments is calculated and presented in the table below.

**Figure 7:**
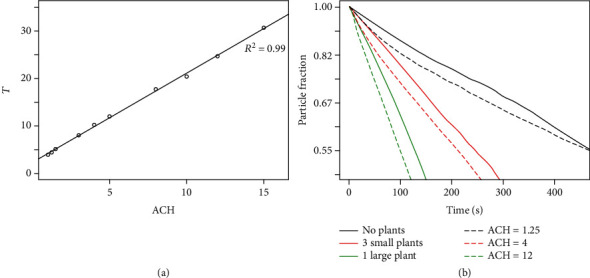
(a) Plot of effect of ACH on the decay constant within the room. (b) Illustrative plots of the reduction in particle counts in the room with time when different plant-based ionizers are used, and when the room is simulated with different ventilation rates.

In general, we note that the use of plant-based ionizers can indeed greatly increase the decay rate from 2 to 4 hr^−1^ to 25 hr^−1^ in the room tested. The decay of particles in the modelled indoor space with varying ventilation rates (ACH) is also calculated and presented in Figure 7. This was done to provide an intuitive comparison of the effectiveness of the tested plant-based ionizers relative to adjustments in ventilation rates of similar indoor environments.

For this simplified generic room, the numerical simulations indicate that increasing the inlet flow velocity from a situation where ACH = 1 to ACH = 15 results in a relatively proportional increase in *τ*, although this might vary with more complex geometries. As a simple comparison, we further note that an equivalent improvement in *τ* from the use of 3 small plants or 1 large plant relative to the no plant experimental setting would require a 3.2× and 9.6× increase, respectively, in the inlet volumetric flow rate (or ACH) of the room, from ACH = 1 to ACH = 5 and ACH = 12. It should be noted that a 9.6× increase in the inlet flow velocity of air into the room is anticipated to be very difficult to implement, whereas the installation of a large plant-based ionizer is comparatively straightforward. Lastly, typical ACH values for various indoor settings are listed in Table 3.

In addition, it is worth noting that the ACH-equivalent values reported here is specifically for 1 *μ*m aerosol. The ACH values measured using tracer gas (i.e., CO_2_), which represents the real air-exchange in the room, tend to overestimate the equivalent ACH for contaminant removal [45]. This is due to the different behaviours of gas and particles (contaminants) which have discrete masses and sizes, and, therefore, more difficult to be purged out. Furthermore, the location of air inlet and outlet (vent) may also affect the contaminant/aerosol removal efficacy (i.e., for the same level of real air-exchange, if the outlet is located near the top of the room/enclosure, it will be more difficult to purge out the contaminants due to the competing effect with gravitational settling).

While ACH-equivalent is a convenient metric to compare the efficacies of various types of plant-based ionizers within the same room/enclosure, it does not provide the air-cleaning capacity of each of these plant-based ionizers. A golden standard to evaluate the efficacy of conventional air-purifiers is the clean air delivery rate (CADR). Although the mechanism for these plant-based ionizers is very different from conventional filter-based purifiers, the aerosol removal efficacy can still be expressed in CADR-equivalent to enable across the board comparison. Table 2 shows the equivalent ACH achieved in a 20 m^3^ room, CADR, and the NAIs concentration for each plant-based ionizer. Evidently, CADR is a convenient metric to estimate the maximum cleaning capacity (room volume) of each plant-based ionizer to be able to achieve a certain ACH value. In addition, as laid out in earlier discussion on the size and number of plant-based ionizers, NAI concentration is a key determining factor in the efficacy of aerosol removal.

## 4. Conclusions

In the context of mitigating COVID-19 transmission via aerosol, this work laid out important guiding principles in deploying plant-based ionizers for application in indoor environments. Under realistic operating conditions, an ACH-equivalent of 12 and CADR-equivalent of 141 ft^3^/minute can be achieved by deploying a proper-sized plant in a poorly ventilated room. The proposed solution using plant-based ionizers is part of a multipronged approach, together with proper safe-distancing, mask-wearing, personal-hygiene, and fomite-disinfecting to further minimize the risk of airborne transmissions.

## Figures and Tables

**Table 1:** Experimental decay constants and ACH for different RH (relative humidity) and enclosure size.

Enclosure	Ionizer(s)	RH	*τ* (hr^−1^)	Equivalent ACH
Poorly ventilated room	Nil	66%	4.5	1.0
Poorly ventilated room	2 small plants	66%	8.8	4.0
Poorly ventilated room	3 small plants	66%	10.2	5.0
Poorly ventilated room	1 large plant	66%	25.7	12.0
Poorly ventilated room	1 coco coir	66%	25.3	12.0
Poorly ventilated room	Nil	95%	2.4	~0
Poorly ventilated room	1 large plant	95%	12.9	5.0
Poorly ventilated room	3 small plants	95%	2.1	~0
Box	Nil	50%	3.9	1.0
Box	1 small plant	50%	30.9	15.0

^∗^Enclosure ACH was experimentally measured using CO_2_ decay data. ^#^CO_2_ ACH for poorly ventilated room and box are 1.6 and 0.04, respectively.

**Table 2:** Equivalent ACH and CADR for various types of plant-based ionizers and their respective NAIs concentration.

Ionizer	Equivalent ACH in 20 m^3^ room	1 *μ*m CADR (ft^3^/minute)	NAI concentration per ionizer^∗^ (/cm^3^)	Maximum volume to maintain at least ACH 6 #
Commercial ionizer	0.1	1.5	3,000	0.4 m^3^
3 small plants	5	60	200,000	17 m^3^
1 large plant	12	141	900,000	40 m^3^
1 coco coir	12	141	900,000	40 m^3^

^∗^NAI concentration was measured 80 cm from source using COM-3200PRO II. ^#^ACH 6 is the minimum recommended by centers for disease control and prevention (CDC) in general hospital rooms.

**Table 3:** Typical ACH values for different indoor settings.

Setting	Typical ACH
Basement	3-4
Kitchen	7-8
Living room	6-8
Office	6-8
Hospital	6-12
Restaurant	8-10
Conference room	8-12

## Data Availability

The data used to support the findings of this study are available from the corresponding author upon request.

## Supplementary Materials

Supplementary 1 (Supplementary movie 1): the plant-based ionizer is turned on, swirl eddies can be observed around the plant-based ionizer.

Supplementary 2 (Supplementary movie 2): the plant-based ionizer was turned on, the NAIs charged the aerosol, which induce opposite charges on the nearby surface/wall and consequently being deposited onto the wall as fomites.
